# Dataset of the land use pattern optimization in Horqin Sandy Land

**DOI:** 10.1016/j.dib.2020.106335

**Published:** 2020-09-24

**Authors:** Wenjie ZHU, Yang GAO, Cuiling SONG

**Affiliations:** a College of Land Science and Technology, China Agricultural University, Beijing 100193, China

**Keywords:** Land use change, Multi-scenario simulation, Ecosystem services, Horqin Sandy Land

## Abstract

This dataset uses downloadable public datasets such as the Harmonized World Soil Database (HWSD) to account for ecosystem services such as net primary productivity (NPP) in Horqin Sandy Land in 2015 through ecological process models. The land use pattern of Horqin Sandy Land under three scenarios in 2025 was obtained by CLUMondo model. Based on the spatial distribution of ecosystem services in Horqin Sandy Land in 2015, the land use under three scenarios in 2025 was used as a variable to obtain the optimal pattern of ecosystem services in Horqin Sandy Land through Netica software. This dataset combines land use simulation with ecosystem service optimization, and can provide reference for decision makers and stakeholders to formulate ecosystem governance policies [1].

## Specifications Table

**Table:** 

Subject	Ecology
Specific subject area	Ecosystem services
Type of data	Table Figure Raster (Geotiff)
How data were acquired	Data: The raw data is mainly downloaded from some public datasets, such as remote sensing data, meteorological data and statistical data. Instruments: ArcMap, CLUMondo, Netica.
Data format	Raw and analyzed
Parameters for data collection	This dataset includes the spatial pattern of the three services: net primary productivity (NPP), crop production (CP), and wind protection and sand fixation (WPSF) in Horqin Sandy Land in 2015, the land use pattern in 2025 under the three scenarios, and a subset of key variables and key states of each level of NPP, CP and WPSF in 2025. Moreover, some parameter settings in CLUMondo are described in detail.
Description of data collection	Analyze and process downloadable spatial datasets and statistical data such as the Harmonized World Soil Database to obtain land use optimization datasets in Horqin Sandy Land.
Data source location	Horqin Sandy Land (118^°^ 35′-123^°^ 30′E, 42^°^ 41′-45^°^ 15′N) is composed of the entire territory of Tongliao City and a part of Chifeng City and Xing'an League in Inner Mongolia Autonomous Region, China.
Data accessibility	The availability of data is described and displayed in this article. Part of the data can be downloaded in the supplementary materials of this article.
Related research article	Zhu, W., Gao, Y., Zhang, H., & Liu, L. (2020). Optimization of the land use pattern in Horqin Sandy Land by using the CLUMondo model and Bayesian belief network. Science of The Total Environment, 139929. https://doi.org/10.1016/j.scitotenv.2020.139929.

## Value of the Data

• The dataset establishes the spatial optimization model of ecosystem services under different land use scenarios to improve human well-being in Horqin Sandy Land.
• The dataset provides a reference for policy makers and stakeholders to realize the sustainable development of Horqin Sandy Land.
• The dataset combines the optimization pattern of ecosystem services with the land use probability surface and can be applied to the areas where ecosystem services are comprehensively improved in the future.
• The dataset provides data support for further research on the trade-off and synergy between the ecosystem services of Horqin Sandy Land.

## 1 Data Description

### 1.1 Land use simulation

The CLUMondo model was used to simulate the land use under three scenarios in 2025, namely historical trend (HT), national planning (NP), and windbreak and sand fixation (WS). Some specific parameters need be input into CLUMondo model, such as land use order, conversion resistance, and conversion matrix of land use.

The land use order indicates the degree to which the land use type can meet the land use demand. The value can be any integer, and a larger value means more supply. 0 means that the land use type does not provide the service. Cultivated land area, forage yield, and forest area were selected to characterize the land use demand of Horqin Sandy Land (Table 1).

**Table 1:** Land use order of CLUMondo model.

Land use demand\Land use type	Cultivated area	Forage yield	Forest area
Cultivated land	1	0	0
Forest	0	0	1
Grassland	0	1	0
Water	0	0	0
Build-up areas	0	0	0
Unused areas	0	0	0

The CLUMondo model has specific parameters, including conversion resistance, conversion matrix of land use and driving factors. Conversion resistance characterizes whether the land use type can change reversibly, and its value is between 0 and 1. The larger the value is, the less likely the land use type changes. Conversion resistance of cultivated land, forest, grassland, water, build-up areas, and unused areas in this dataset are 0.8, 0.9, 0.9, 0.9, 1.0, and 0.8, respectively. The conversion matrix of land use indicates whether the two land use types can be converted to each other. Its value is 0 or 1. 0 means no conversion is allowed, and 1 means conversion is allowed. Since build-up areas is not easily converted into cultivated land, forest, grassland and water, this dataset imposes restrictions on the conversion of construction land, assuming that other land use types can be converted to each other. Six types of driving factors for land use change are selected, including climate factors, soil property factors, topographic factors, vegetation factors, socioeconomic factors, and location factors. The detailed settings can be obtained from related article [1].

Using the logistic regression tool embedded in CLUMondo model to test the significance of non-collinearity driving factors. Since the CLUMondo model can only support linear regression of 7 driving factors at most, the 7 most significant driving factors of each land use type are selected for linear regression.

By setting different land use demand (Table 2), three scenarios are generated in CLUMondo model. In the HT scenario, based on the land use of Horqin Sandy Land in 2005 and 2015, linear interpolation was used to calculate the land use demand from 2005 to 2015. In the NP scenario, the forest area of Horqin Sandy Land from 2015 to 2020 is calculated based on an annual increase of 0.4%. In the WS scenario, the forest and grassland areas increase by 5.57% each from 2015 to 2020. Finally, the above trends were extended to 2025 by linear interpolation.

**Table 2:** Land use demand from 2015 to 2025 in Horqin Sandy Land.

	Cultivated area (km^2^)			Forage yield (10,000 tons)			Forest area (km^2^)		
Scenario\Year	HT	NP	WS	HT	NP	WS	HT	NP	WS
2015	34618.00	34618.00	34618.00	4203.42	4203.42	4203.42	12880.00	12880.00	12880.00
2016	34688.40	34688.40	34688.40	4196.36	4250.25	4196.36	12876.90	13023.48	12931.52
2017	34758.80	34758.80	34758.80	4189.30	4297.07	4189.30	12873.80	13166.97	12983.25
2018	34829.20	34829.20	34829.20	4182.24	4343.90	4182.24	12870.70	13310.45	13035.18
2019	34899.60	34899.60	34899.60	4175.18	4390.73	4175.18	12867.60	13453.93	13087.32
2020	34970.00	34970.00	34970.00	4168.12	4437.55	4168.12	12864.50	13597.42	13139.67
2021	35040.40	35040.40	35040.40	4161.06	4484.38	4161.06	12861.40	13740.90	13192.23
2022	35110.80	35110.80	35110.80	4154.00	4531.20	4154.00	12858.30	13884.38	13245.00
2023	35181.20	35181.20	35181.20	4146.95	4578.03	4146.95	12855.20	14027.87	13297.98
2024	35251.60	35251.60	35251.60	4139.89	4624.86	4139.89	12852.10	14171.35	13351.17
2025	35322.00	35322.00	35322.00	4132.83	4671.68	4132.83	12849.00	14314.83	13404.57

The CLUMondo model was set with the above parameters and data input. The land use of Horqin Sandy Land in 2025 under three scenarios were listed in Supplementary.

### 1.2 Optimization of ecosystem service pattern

**Fig. 1:**
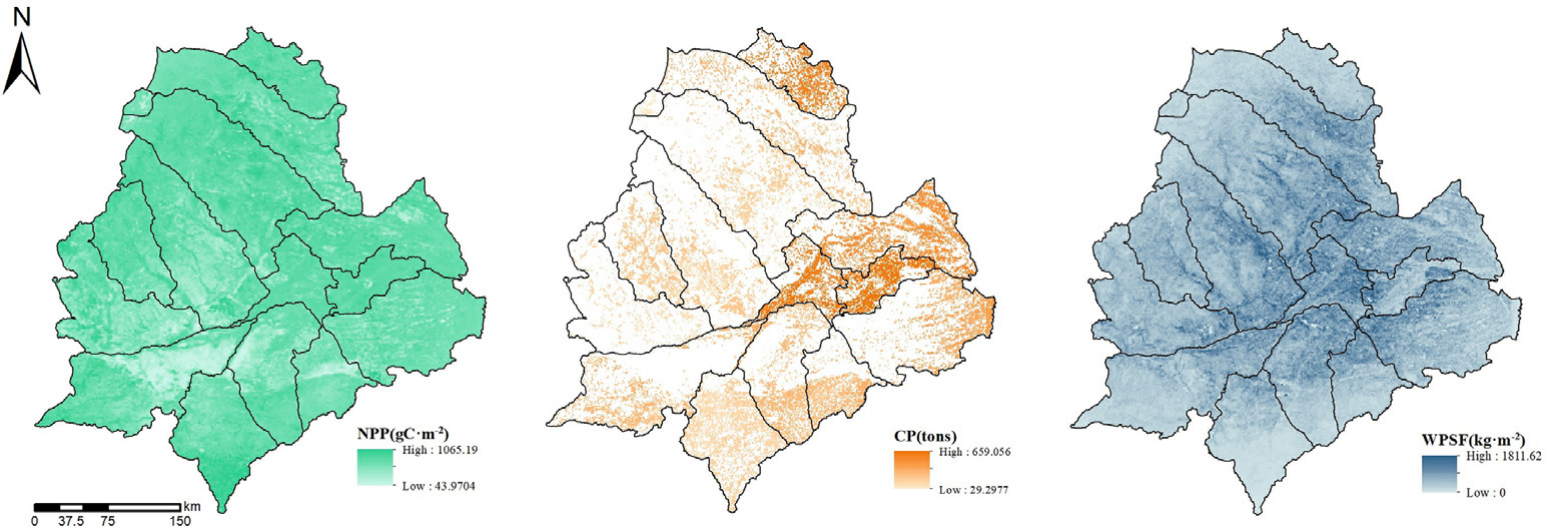
NPP, CP and WPSF of Horqin Sandy Land in 2015.

The Carnegie–Ames–Stanford approach (CASA) model, NDVI characterization, and Revised Wind Erosion Equation (RWEQ) model were used to calculate NPP, CP and WPSF in Horqin Sandy Land in 2015, and rasterized them in ArcMap (Fig. 1).

By establishing a Bayesian brief network (BBN) composed of ecosystem services and impact factors in 2015, using land use in 2025 as a variable variable, the probability of different levels of the three ecosystem services is determined and rasterized in ArcMap. The data in raster format was listed in the Supplementary.

## 2 Design, Materials and Methods

### 2.1 Land use simulation

The CLUMondo model was used to simulate the land use under three scenarios. The specific parameters are explained as follows:

Exclusion layers: The national nature reserve in Horqin Sandy Land was regarded as the exclusion layer of CLUMondo model, which was not involved in the land use simulation and kept the land use type unchanged.

Land use demand: The land use demand of CLUMondo model was represented by cultivated land area, forage yield and forest area.

Parameters of land use conversion: Land use order, conversion resistance, and conversion matrix.

Driving factors: considering the actual situation of the study area and previous researches [2], [3], six types of driving factors were selected, namely, climate, soil property, topographic, vegetation, location and socioeconomic factors.

### 2.2 Quantification of ecosystem services

#### 2.2.1 CP

With the total crop production obtained from the statistical yearbooks of the relevant counties or banners in Horqin Sandy Land, the spatialization raster can be calculated according to the NDVI of the cultivated land in the study area. The data in raster format was listed in the Supplementary.

#### 2.2.2 NPP

NPP was calculated using an improved CASA model combined with the distribution characteristics of terrestrial vegetation in China [4], especially the possible efficiency of different vegetation types under ideal conditions. The data in raster format was listed in the Supplementary.

#### 2.2.3 WPSF

The RWEQ was used to calculate the potential wind erosion and the actual wind erosion. The difference between them was used to characterize WPSF. RWEQ was composed of factors that affect the wind erosion process, including weather factor (WF), soil erodibility factor (EF), soil crust factor (SCF), soil roughness factor (K_0_) and vegetation factor (C) [5]. Each factor was calculated and spatialized in ArcMap. WF is related to wind speed, multi-year average soil moisture factor and snow cover factor. EF is related to the content of each component in the soil. SCF is related to the clay content and organic matter content. K_0_ is related to the roughness of soil ridges and surface undulation. C varies with vegetation types.

The detailed calculation method can be obtained from related article [1].

### 2.3 Ecosystem services optimization

BBN was built with Netica software. NPP, CP and WPSF were selected as child node, and influence factors of them were selected as parent node. BBN is constructed based on expert knowledge and the causal relationship between variables. The variables in Horqin Sandy Land in 2015 were used as sample data to train the established BBN to determine the conditional probability table (CPT). Based on the verification of the CPT, the land use in 2025 is taken as a variable to obtain the probability of each level of NPP, CP and WPSF in 2025.

Then, the key variables and the key states were further determined to optimize the ecosystem service pattern [6]. The sensitivity analysis method of Variance Reduction (VR) was used to determine the key variables. The VR value can be calculated as follows:

(1) VR=V(Q)−V(Q|F)

(2) $$\;\;V\left(Q\right)=\sum_{q}p\left(q\right)\times {\left[X_{q}-E\left(Q\right)\right]}^{2}$$

(3) $$V\left(Q|F\right)=\sum_{q}p\left(q|f\right)\times {\left[X_{q}-E\left(Q|f\right)\right]}^{2}$$

where *VR* is variance reduction; *V*(*Q*) is the variance of ecosystem services *Q; V*(*Q|F*) is the variance of ecosystem service *Q* under variable *F; q* is the number of states of ecosystem services *Q; f* is the number of states of the variable *F; X_q_* is the value of state *Q; E*(*Q*) is the expectation of the ecosystem service *Q; E*(*Q|F*) is the expectation of ecosystem service *Q* under variable *F*.

After determining the key variables, the key state could be determined by calculating the conditional probability. The calculation formula of conditional probability is as follows:

(4) $$P\left(A|B\right)=\frac{P\left(AB\right)}{P\left(B\right)}$$

where *P*(*A|B*) is the probability of child node *A* occurring if parent node *B* occurs; *P*(*AB*) is the probability of simultaneous occurrence of parent node *B* and child node *A; P*(*B*) is the probability that the parent node B occurs.

The data of ecosystem services optimization in raster format was listed in the Supplementary.

## Declaration of Competing Interest

The authors declare that they have no known competing financial interests or personal relationships which have, or could be perceived to have, influenced the work reported in this article.

## Appendix Supplementary materials

Image, application 1

## Footnotes

Supplementary material associated with this article can be found, in the online version, at doi:10.1016/j.dib.2020.106335.
