# Supplementary figures and images for: Dataset of the land use pattern optimization in Horqin Sandy Land
Source: Data Brief. 2020 Sep 24;33:106335. doi: 10.1016/j.dib.2020.106335 (PMC7530146; doi:10.1016/j.dib.2020.106335)

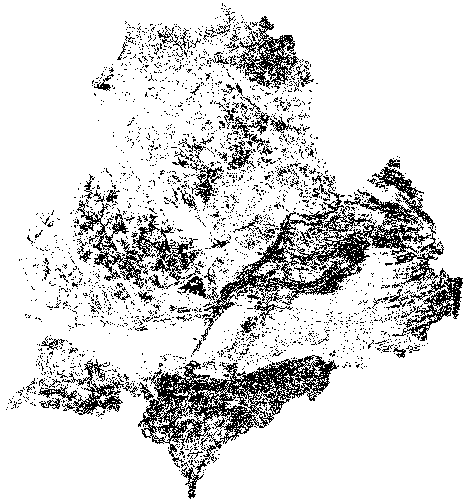

Supplement: Supplementary file 1 [file mmc1.zip › Subsets of ecosystem services optimization/CP_higher.tif]

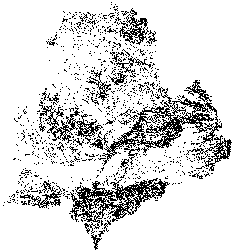

Supplement: Supplementary file 1 [file mmc1.zip › Subsets of ecosystem services optimization/CP_higher.tif.ovr]

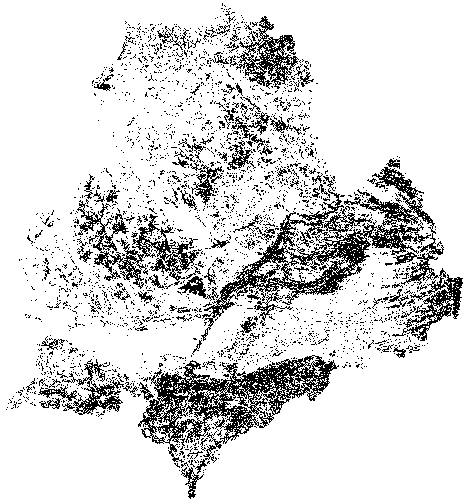

Supplement: Supplementary file 1 [file mmc1.zip › Subsets of ecosystem services optimization/CP_highest.tif]

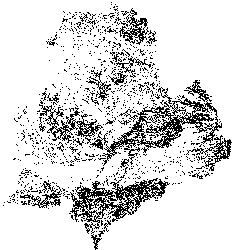

Supplement: Supplementary file 1 [file mmc1.zip › Subsets of ecosystem services optimization/CP_highest.tif.ovr]

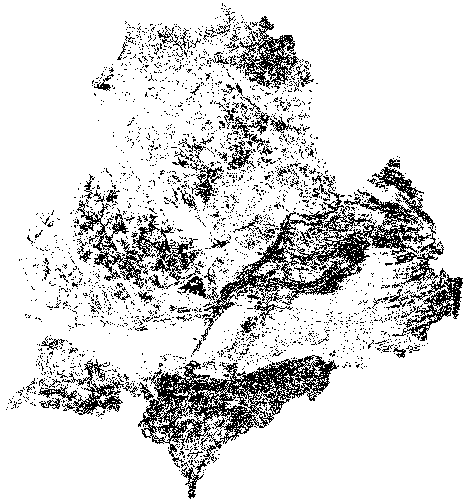

Supplement: Supplementary file 1 [file mmc1.zip › Subsets of ecosystem services optimization/CP_low.tif]

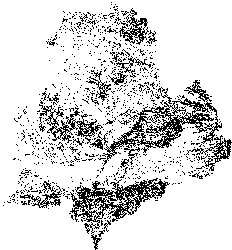

Supplement: Supplementary file 1 [file mmc1.zip › Subsets of ecosystem services optimization/CP_low.tif.ovr]

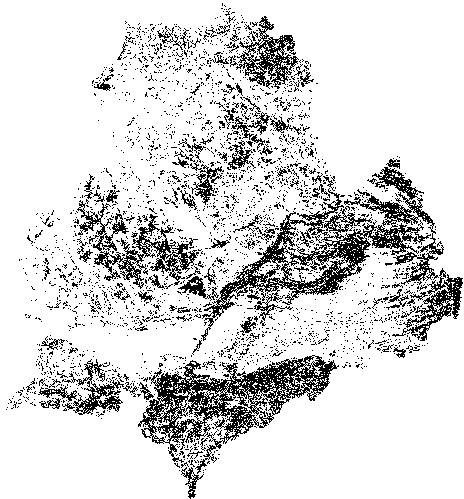

Supplement: Supplementary file 1 [file mmc1.zip › Subsets of ecosystem services optimization/CP_medium.tif]

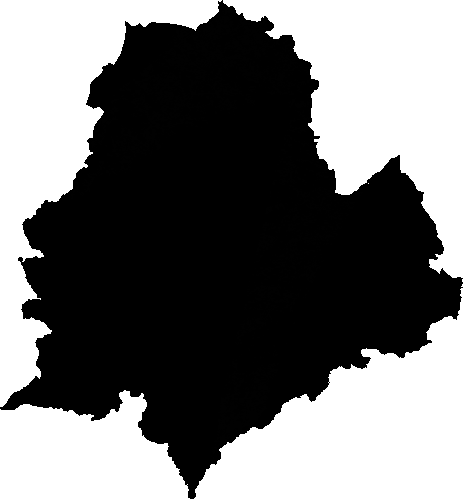

Supplement: Supplementary file 1 [file mmc1.zip › Subsets of ecosystem services optimization/NPP_higher.tif]

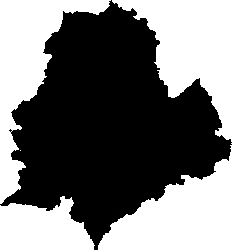

Supplement: Supplementary file 1 [file mmc1.zip › Subsets of ecosystem services optimization/NPP_higher.tif.ovr]

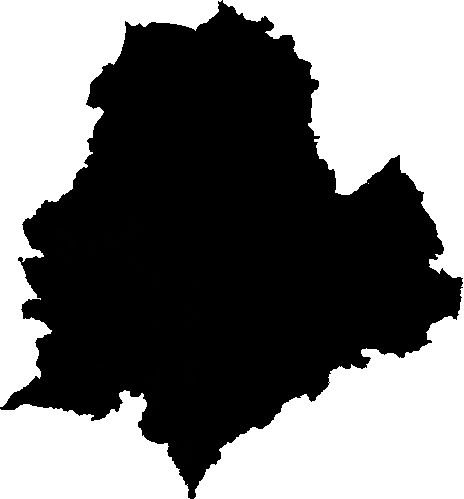

Supplement: Supplementary file 1 [file mmc1.zip › Subsets of ecosystem services optimization/NPP_highest.tif]

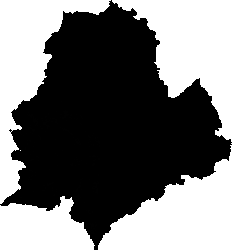

Supplement: Supplementary file 1 [file mmc1.zip › Subsets of ecosystem services optimization/NPP_highest.tif.ovr]

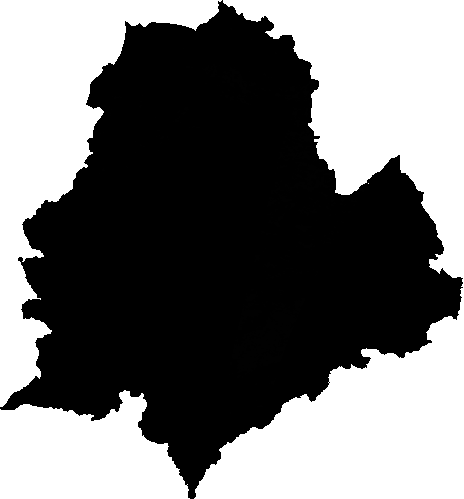

Supplement: Supplementary file 1 [file mmc1.zip › Subsets of ecosystem services optimization/NPP_low.tif]

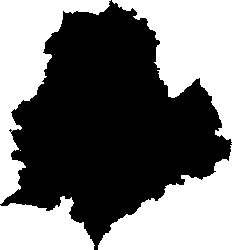

Supplement: Supplementary file 1 [file mmc1.zip › Subsets of ecosystem services optimization/NPP_low.tif.ovr]

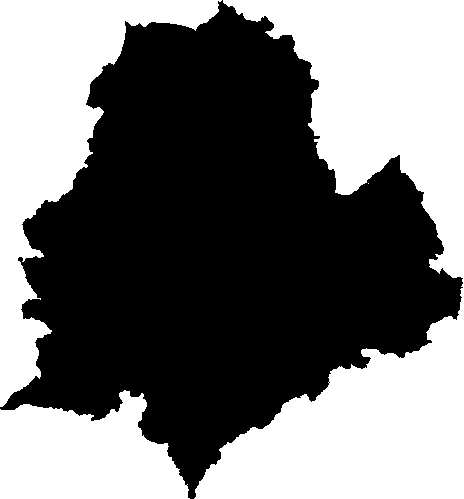

Supplement: Supplementary file 1 [file mmc1.zip › Subsets of ecosystem services optimization/NPP_medium.tif]

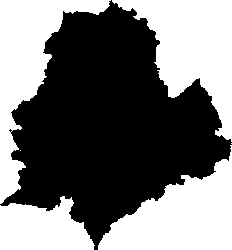

Supplement: Supplementary file 1 [file mmc1.zip › Subsets of ecosystem services optimization/NPP_medium.tif.ovr]

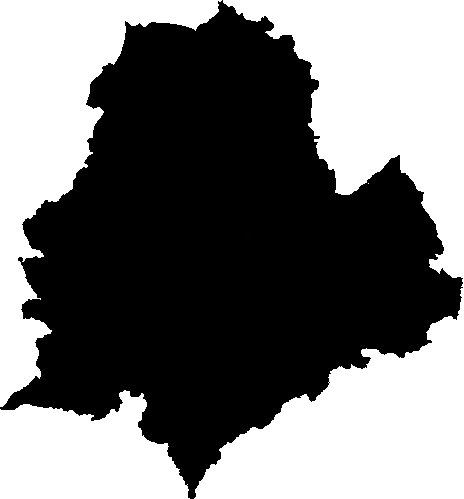

Supplement: Supplementary file 1 [file mmc1.zip › Subsets of ecosystem services optimization/WPSF_higher.tif]

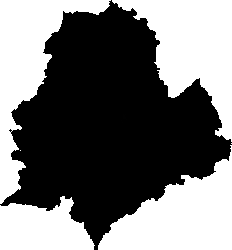

Supplement: Supplementary file 1 [file mmc1.zip › Subsets of ecosystem services optimization/WPSF_higher.tif.ovr]

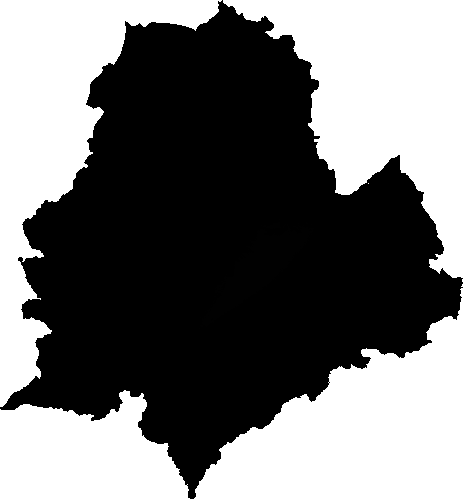

Supplement: Supplementary file 1 [file mmc1.zip › Subsets of ecosystem services optimization/WPSF_highest.tif]

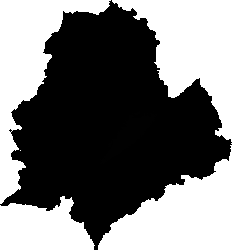

Supplement: Supplementary file 1 [file mmc1.zip › Subsets of ecosystem services optimization/WPSF_highest.tif.ovr]

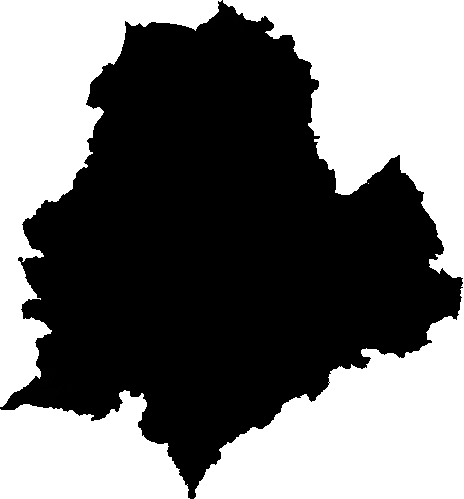

Supplement: Supplementary file 1 [file mmc1.zip › Subsets of ecosystem services optimization/WPSF_low.tif]

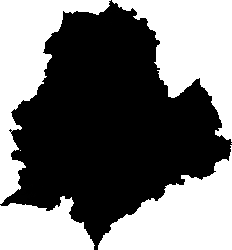

Supplement: Supplementary file 1 [file mmc1.zip › Subsets of ecosystem services optimization/WPSF_low.tif.ovr]

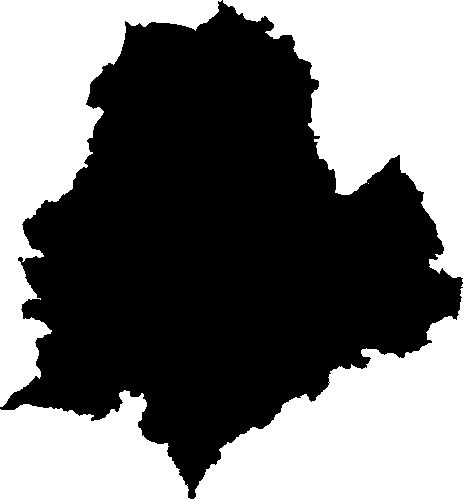

Supplement: Supplementary file 1 [file mmc1.zip › Subsets of ecosystem services optimization/WPSF_medium.tif]

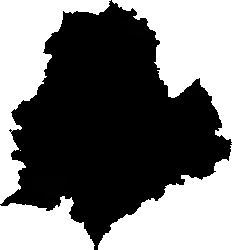

Supplement: Supplementary file 1 [file mmc1.zip › Subsets of ecosystem services optimization/WPSF_medium.tif.ovr]
